# Augmenting healthcare systems for pandemic preparedness: a Lean Six Sigma perspective

**DOI:** 10.1093/intqhc/mzag071

**Published:** 2026-05-22

**Authors:** Utkarsh Chadha, Artem Kushnirenko, Darren Charles Fernandes, Krisha Bhavesh Patel, Norah Jean Crothers, Harpreet Singh, Mete Isiksalan, Ibrahim Nasir, Stephen Armstrong, Kamran Behdinan

**Affiliations:** Department of Mechanical and Industrial Engineering, University of Toronto, Toronto, ON M5S 3G8, Canada; Department of Medical Biophysics, Temerty Faculty of Medicine, University of Toronto, Toronto, ON M5G 2C4, Canada; Arthur and Sonia Labatt Brain Tumour Research Centre (BTRC), Peter Gilgan Centre for Research and Learning, SickKids—The Hospital for Sick Children, Toronto, ON M5G 0A4, Canada; Department of Mechanical and Industrial Engineering, University of Toronto, Toronto, ON M5S 3G8, Canada; Department of Civil and Mineral Engineering, University of Toronto, Toronto, ON M5S 1A4, Canada; Health Studies Program, University College, University of Toronto, Toronto, ON M5S 3H7, Canada; Department of Mechanical and Industrial Engineering, University of Toronto, Toronto, ON M5S 3G8, Canada; The Edward S. Rogers Sr. Department of Electrical & Computer Engineering, University of Toronto, Toronto, ON M5S 3G8, Canada; The Edward S. Rogers Sr. Department of Electrical & Computer Engineering, University of Toronto, Toronto, ON M5S 3G8, Canada; Department of Mechanical and Industrial Engineering, University of Toronto, Toronto, ON M5S 3G8, Canada; AMGI Management Group Inc., Toronto, ON, Canada; Advanced Research Laboratory for Multifunctional Lightweight Structures, Department of Mechanical and Industrial Engineering, University of Toronto, Toronto, ON M5S 3G8, Canada

**Keywords:** Lean Six Sigma, Pandemic Preparedness, Healthcare systems, Healthcare management, Quality improvement, Healthcare resilience, Hospital operations

## Abstract

**Background:**

Past global healthcare crises have exposed vulnerabilities in healthcare systems, including inefficiencies in hospital operations, delayed response times, and overburdened infrastructure. Traditional hospital systems built for routine care were sometimes not resilient or adaptable in the face of such crises, resulting in global failures. This narrative review examines how Lean Six Sigma (LSS) principles can be incorporated into conventional hospital operations to enhance pandemic preparedness by building the resilience of hospital infrastructure, streamlining processes during time-sensitive situations, and improving waste reduction, all while being adaptable and sustainable.

**Methods:**

This narrative review synthesizes literature using Coronavirus Disease 2019 (COVID-19) as a benchmark to evaluate hospital response strategies, failures, and factors contributing to failure, including the critical assessment of ethical disruptions, operational weaknesses, and healthcare business models. LSS principle applications, i.e. DMAIC, Value Stream Mapping, SIPOC, FMEA, and Control Charts, were explored for facilitating efficient care, crisis response, and policy integration. Various case studies were used to support the comparative analysis and insights.

**Results:**

The literature indicates that adoption of LSS tools in the most vulnerable aspects of healthcare, including patient triage, supply chain optimization, and controlling and reducing mortality, has been associated with measurable improvements. Most importantly, integrating data-driven LSS resulted in enhanced surge responsiveness and ethical compliance within national healthcare frameworks and policies. However, despite its efficacy, there are institutional barriers like capital constraints, resistance to change, data inconsistencies, and a lack of legislative frameworks that impede widespread LSS adoption.

**Conclusion:**

LSS offers an adaptable and scalable methodology to re-engineer conventional hospital operations and pandemic preparedness. The emphasis on ‘kaizen’ (continuous improvement), data-informed decision making, and focus on precision aligns with the needs of healthcare systems as revealed by recent crises. To unlock the potential for preparedness, healthcare systems and legislation must focus on institutionalizing LSS across public and private sectors through strategic investment, education, and cross-sector collaborations. This review provides a comprehensive framework for policymakers, governments, epidemiologists, doctors, and hospital business managers for building resilient, efficient, and pandemic-ready hospitals.

## Introduction

The COVID-19 pandemic exposed faulty healthcare and business models and led to widespread systemic failure [1–4]. Increased patient intake added more pressure on the healthcare system to identify, separate, and treat the critical patients appropriately [5, 6]. On-ground healthcare workers, who navigated the huge influx of patients and their associated medical complexities, faced immense pressure [7, 8]. Moreover, immunocompromised individuals and people with comorbidities had a higher mortality rate from COVID-19, worsening system overload [9–11].

Systemic failures also arose from differences in hospital management structure. For example, in Germany, healthcare funding varies by region or the type of funding/ownership. The Bavaria region has more public hospitals, while Berlin has more private hospitals [12]. As a result, hospital pandemic responses may be entirely different [13–15]. These differences may cause communication gaps in essential protocols.

Another consequence of the pandemic was constant uncertainty. In many countries, mental health issues worsened, and widespread depression arose due to lockdowns and isolation. Financial uncertainty arose as well. Alongside affordability, the number of well-equipped hospitals is critical to managing patient influx [16]. The USA has one of the lowest numbers of public hospitals in OECD countries, around 4.32 public hospitals per million people [17]. Moreover, only the United Kingdom’s General hospitals, about 38%, were properly equipped to handle COVID-19 protocols [17].

The uncertainties and mismanagement during the COVID-19 crisis that led to healthcare system failure inspired this work. This review emphasizes how human error reduction and precision is crucial to pandemic preparedness. This is where Lean Six Sigma (LSS), a philosophy that combines waste reduction, precision, and quality, can come into play. LSS would complement hospital infrastructure by managing patient intake, resources, and care, even in highly uncertain periods like a pandemic [18]. LSS can enable smooth adaptation during surges and improve patient categorization and discharge [19]. This review provides an overview of potential LSS strategies to achieve these goals.

## Materials and methods

This article is structured as a narrative review synthesizing literature on healthcare system failures observed during the COVID-19 pandemic and the application of LSS for pandemic preparedness. Articles were identified through searches of PubMed, Scopus, Google Scholar, and major public health repositories between 2020 and 2025, supplemented by policy documents and relevant grey literature.

Included sources examined healthcare system performance, ethical and operational disruptions, and LSS-based interventions relevant to crisis response. The literature was thematically synthesized across operational, managerial, ethical, and policy domains to generate an integrated analytical narrative.

## Results

### Ethical and legal foundations of healthcare: limitations during the pandemic

Four of the fundamental principles of healthcare, i.e. autonomy, beneficence, non-maleficence, and justice, ensure patient rights, equitable care, and ethical decision-making in medical practice [20]. During COVID-19, these principles eroded. Bureaucracy and resource limits hindered ethical responses [21].

Autonomy underpins the right of individuals to make voluntary healthcare decisions, but was subordinated to collective welfare during the pandemic. Quarantines, visitation bans, and vaccine mandates aimed at containing the COVID-19 virus raised concerns about state interventions into personal medical choice and the severity of lockdowns [22, 23]. The impacts of these lockdown measures were notably uneven. The pandemic vividly illustrated how social vulnerabilities intersect with clinical and institutional constraints. For example, low-income regions faced delayed vaccine access and inconsistent messaging [24].

Beneficence and non-maleficence require healthcare providers to act in the best interest of patients and avoid doing harm. However, overwhelmed hospital systems, staff shortages, and delays complicated triage decisions [22]. Justice, the equitable distribution of healthcare resources, became particularly contentious as ventilators, ICU beds, and vaccines were rationed. In the USA, triage criteria varied by state, producing unequal outcomes [25]. The erosion of core ethical principles during the COVID-19 crisis serves as an indicator of systemic unpreparedness.

The recent pandemic challenged not only the moral architecture of the healthcare system but also the logistics of delivery. Legal frameworks regarding preparedness were not adequately elaborated on or enforced. For example, Canada’s *Pandemic Prevention and Preparedness Act* (Bill C-293) and the WHO’s Pandemic Treaty draft aimed to emphasize coordination, equity, and risk mitigation. Bill C-293 requires the Minister of Health to consult with Indigenous communities, provinces, and international bodies to develop a pandemic response plan [26]. However, it failed to translate these aspirations into concrete mechanisms. It does not define specific protocols for triage, patient rights under lockdown, or metrics ensuring equitable resource distribution. Similarly, the WHO treaty emphasizes global access to vaccines and surveillance systems but does not include specific legal instruments to protect or enforce ethical norms at the clinical level [27].

This disconnect between ethics and execution makes healthcare systems structurally vulnerable, leaving frontline providers to react as challenges arise. In the absence of triage frameworks, healthcare providers were left to make decisions on a case-by-case basis. In Italy, early in the pandemic, ICU triage guidelines suggested that ventilator access should prioritize younger or less comorbid patients [28]. These examples illustrate that policy-level commitments to equity and human rights remain largely rhetorical unless tied to enforceable, measurable standards. A deeper analysis of legal frameworks is discussed in Supplementary Appendix A.

### Benchmarking COVID-19: failure cascade

Preoccupied with the virus epidemiology, few institutions were technologically prepared to withstand the operational shock that followed. The failures during COVID-19 were not merely clinical, but strategic and managerial. The most prominent failure was fragmented coordination between government bodies and care providers. Government responses were marred by ineffective recommendations and widespread misinformation, leading to disjointed public behavior and response. For example, the conflicting data control and siloed communication between the Governor’s office and municipal health authorities in New York hindered resource deployment and public messaging, leading to duplication of efforts [29].

At the hospital level, operational structures designed primarily for routine service efficiency rather than crisis adaptation contributed to record-high emergency department wait times, missed cancer treatment targets, and procedural backlogs during the pandemic [30]. Financial strain profoundly affected both private and public healthcare segments, undermining operational functionality [31, 32]. American hospitals, for example, faced severe financial challenges when non-emergency care was suspended in order to prioritize COVID-19 patients [33]. Frontline professionals experienced severe burnout [34]. As a result of these struggles, ambulatory care services saw over 1.2 million job losses in the USA by April 2020 [35]. Economically vulnerable populations, including those living below the poverty line or those who lost employment during the pandemic, suffered disproportionately due to insufficient support mechanisms [36].

In the digital realm, hospitals encountered significant challenges related to data management with cybersecurity breaches compromising patient records and operational continuity. These issues were further amplified by legal and institutional barriers to data sharing, which hindered international collaboration and slowed the pace of coordinated research and response efforts [33, 36].

This cascade of failures was reflected in many other industries. The key difference, however, is the healthcare industry’s frontline role in public welfare protections, necessitating additional regulations and ethical conduct. The failure here caused not only a profound wastage of resources but also a devastatingly significant loss of life, an outcome that could have been mitigated through the implementation of streamlined, data-driven process optimization methodologies. Without adaptive capacity and workforce resilience, healthcare systems will continue to buckle under future crises. The pandemic should be regarded not just as a clinical catastrophe, but as a comprehensive failure, and thus a motivation for refinement. LSS could have served as a valuable framework to enhance operational efficiency, reduce variability, and ensure resource optimization, thereby improving both patient outcomes and system resilience [37, 38].

The systemic weaknesses faced by various countries were unique to their supply, business models, and government funding or support. An analysis of case studies indicates systemic failures such as medical understaffing, lack of resources/infrastructure, diagnostic failure, governmental failure, and economic issues [38]. Table 1 comprehensively outlines several countries with systemic weaknesses that led to an exacerbated mortality rate.

**Table 1.** Case studies of systemic weaknesses observed during COVID-19 pandemic

Country	Systemic weakness	Type of weakness	Impact	Mitigation strategies	Reference
USA	Poor PPE stockpiles, hospital bed shortages	Stockpiling, infrastructure	Overcrowded hospitals lead to delayed treatments and increased mortality	International aid for supplying essential PPE stockpiles or products, and the establishment of field hospitals	[36]
Italy	Overwhelmed healthcare infrastructures leading to failure	Infrastructure	Hospitals exceeded capacity, resulting in higher mortality rates	Implementation of field hospitals and expansion of hospital capacity for quicker separation and prioritization of patients	[39]
Canada	Nursing shortages	Human resource	Increased workload leading to personnel burnout and some emergency department closures	Recruitment drives, easing policies and licensing for immigrant doctors or nurses, and incentives to retain nursing staff	[40]
India	Inadequate oxygen supply	Infrastructure	Severe shortages are causing preventable deaths	Ramp-up of oxygen production and importation of supplies	[41]
Nigeria	Insufficient healthcare funding	Economic	Limited resources for patient care and protective measures	Increased government funding and international support	[42]
Brazil	Inconsistent public health messaging	Governance	Public confusion and non-compliance with health directives	Centralized communication strategies and community engagement	[41]

### Applying Lean Six Sigma for pandemic preparedness

#### LSS and healthcare

**Figure 1:**
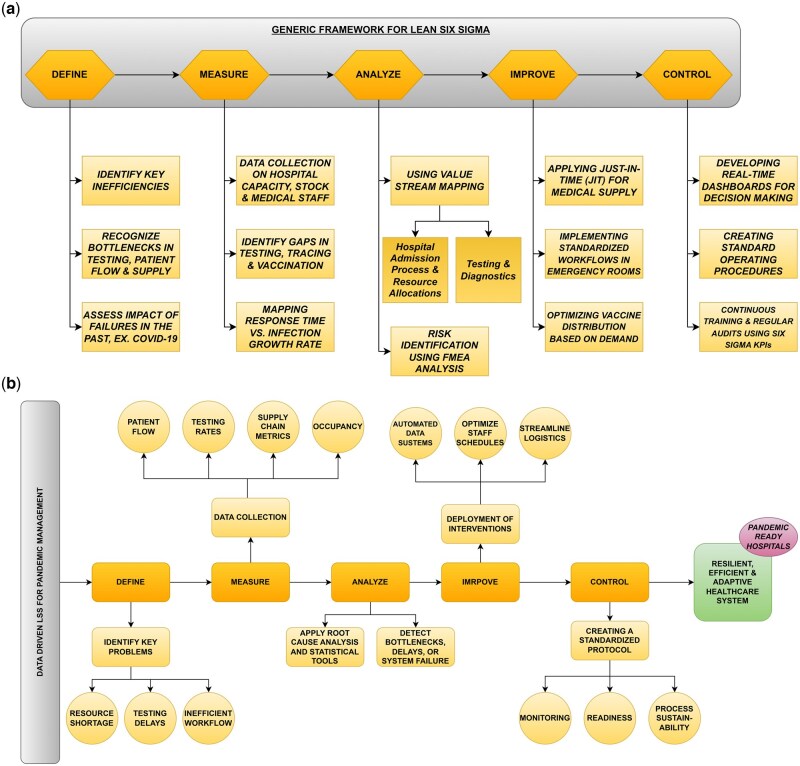
Lean Six Sigma framework: (**a**) Generalized framework for the medical industry. Partial information adapted from [18, 19]. (**b**) Data-driven Lean Six Sigma approach for a pandemic-ready hospitals.

LSS is meant to improve operational efficiency and outcomes by integrating the key methodologies in its name: Lean and Six Sigma [38]. “Lean” uses the DMAIC (Define, Measure, Analyze, Improve, Control) cycle, as shown in Fig. 1a. DMAIC focuses on waste elimination by identifying and removing non-value-added activities (e.g. excessive patient movement, redundant administrative tasks) to streamline healthcare processes. Six Sigma reduces process variability, employing data-driven methods and statistical tools to minimize inconsistencies and defects in healthcare delivery. This ensures consistent outcomes. Kaizen, meaning continuous improvement, complements LSS. Kaizen encourages the ongoing refinement of products, services, and processes. In a healthcare context, this means regular reviews and the optimization of patient care resources.

Applying LSS to healthcare requires additional techniques to honor core aspects of medicine [37]. Most importantly, focusing on the patient (customer) by addressing their needs ensures that process improvements directly translate into better outcomes and experiences. In order to achieve enhanced patient care, LSS uses techniques such as Voice of Customer (VOC). Another crucial aspect of the medical LSS framework is promoting data-driven decision-making. LSS relies heavily on measurement and analysis (e.g. control charts, regression analyses) to guide decision-making and track the effectiveness of changes.

These methodologies and techniques will benefit hospitals in a number of ways [43, 44]. LSS most notably can streamline hospital operations and reduce waste, especially by using tools such as SIPOC (Suppliers, Inputs, Process, Outputs, Customers) and value stream mapping, both of which reduce redundant care and delays. Furthermore, LSS analysis of workload variability and process bottlenecks will allow hospital administration to distribute staff and equipment more efficiently. Root-cause analysis diagrams can aid in the appropriate allocation of resources. Lastly, inefficiencies in procurement and inventory control can be addressed using tools such as control charts and Just-in-Time delivery, a Lean concept. Hospitals could thus ensure that medical supplies are sufficiently stockpiled for crisis scenarios.

#### Data-driven decision-making: LSS for predictive analytics

LSS with predictive analytics can transform how hospitals make strategic decisions. To ensure continuous data for process improvement, DMAIC is enhanced with machine learning and real-time monitoring. By embedding predictive models into the Analyze and Improve phases of the DMAIC cycle (Fig. 1b), organizations can forecast future scenarios and identify potential disruptions [45]. Predictive modelling integrated with control charts, for example, could help forecast ICU bed occupancy rates, oxygen demand, or PPE stock depletion based on infection trends. These insights allow hospitals to simulate surge scenarios, optimize staffing schedules, and implement early triage protocols. LSS tools like Failure Mode and Effects Analysis (FMEA) can be informed by predictive inputs to identify high-risk failure points in emergency workflows before they occur [46].

One Italian hospital applied the DMAIC cycle to assess the impact of the pandemic on the length of stay in the emergency department. By integrating LSS with Machine Learning (ML) models such as Random Forest and Decision Trees, their study found key predictors of bottlenecks like age, mode of arrival, and discharge type. The results showed that patients’ length of stay increased during the pandemic. This underscores the importance of LSS integrated with ML models as a means of predicting latent risk points and enabling early triage and capacity planning [47].

Integrating predictive analytics into LSS creates a feedback loop where control charts are no longer a retrospective tool. With predictive overlays, control charts become early-warning systems that allow healthcare managers to anticipate threshold breaches and take preemptive action. Data-driven LSS can help ensure response measures are not only efficient but also anticipatory. Real-world examples in Table 2 showcase the successful integration of LSS methodologies and highlight significant policy and business benefits.

**Table 2.** Case studies of LSS implementation in healthcare

Case	Type	Context and challenge	LSS phases and implementation					Key outcomes	Reference
			D	M	A	I	C		
Surgical instrument sterilization	Hospital-based implementation	Reduce sterilization errors; improve patient safety, reduce costs, enhance staff satisfaction	Goal to reduce errors and boost compliance	Data on accuracy and inefficiencies.	Root cause + process mapping.	Staff training, SOPs, redesigned workflow.	Ongoing audits and monitoring.	↓ Sterilization errors ↑ Patient safety ↓ Costs ↑ Staff satisfaction	[48]
3D printing of medical implants	Hospital-based implementation	Improve the quality of orthopedic implants via quality by design (QbD) in additive manufacturing	Enhance implant quality via systematic design.	Assessed variability and defects.	Root causes: material issues, machine settings.	QbD integration, optimized design, and parameters.	Rigorous QA and aligned documentation.	↑ Implant quality and reliability ↑ Regulatory readiness ↑ Investor confidence	[49]
Unplanned surgery cancellations	Administrative	Reduce cancellation rates affecting satisfaction, revenue, and efficiency	Objective to reduce cancellations.	Baseline data: frequency and causes.	Process mapping + root cause.	Checklists, better communication, and resource scheduling.	Cyclic monitoring to sustain gains.	↓ Cancellations ↑ Patient satisfaction ↑ Resource efficiency	[50]
Passive immunization project (hospital system)	Governmental, public welfare	COVID-19 response enhancement and routine care optimization	Identified bottlenecks in immunization processes using Voice of Customer (VOC), to identify and prioritize solving areas of concerns like reduced wait time, safer environment, etc.	Used fishbone diagram and data collection to evaluate factors like shared facilities and staff overload.	Used Kano model to classify dissatisfaction drivers, like, long queues, data delays, etc.	Applied Kaizen with 5-whys analysis, using which the solutions were prioritized by costs, impact and feasibility (i.e., shifting OPDs, teleclinic setup, etc.)	Plans included responsibility matrices and monitoring timelines.	↑ Safety ↑ Patient Satisfaction ↑ Optimized staff utilization ↓ Wait times	[37]
COVID-19 testing and call center (green belt project)	Telemedicine	Testing capacity expansion and managing call centers efficiently. Researchers used the University of Michigan’s Green Belt Course to solve the problem.	Identified critical issues in testing and call center workflows coordination	Collected data on current testing data capacities and call handling times to evaluate the current delays.	Identifying redundancies in the workflow, root causes, and data entry inefficiency	Streamlining patient intake and test scheduling, resource reallocation	Standardizing the reporting procedures, dashboards for daily tracking, and training staff to maintain the new workflows.	↑ Testing capacity ↑ Call center operation efficiency ↑ Call volume handling ↓ Data errors ↓ Delays	[44]
Netherlands—national LSS healthcare programs	National level	Balancing long-term efficiency with flexibility and resilience in healthcare.	Identified goals of cost reduction and efficiency improvement through LSS initiatives.	Assessed performance metrics related to cost, quality, and operational efficiency.	Evaluated the impact of LSS on system adaptability and crisis response capabilities.	Recognized the need to balance efficiency with resilience; suggested integrating flexibility into LSS frameworks.	Recommended ongoing monitoring to ensure that efficiency gains do not compromise system adaptability.	↑ Cost efficiency ↑ Streamlined Processes ↓ Flexibility ↓ Resilience	[19]

↑ indicates increase/improvement ↓ indicates decrease/reduction.

### Hospital operations and business strategy: building sustainable models

The failures caused by COVID-19 make a strong case for effective hospital management. Effective management is required to guarantee adequate resources, reduce patient mortality rates, and safeguard healthcare workers. Empirical data from many countries demonstrate the catastrophic effects of weak systems [5, 12, 16]. For instance, hospital wait time for obtaining results from diagnostic tests caused delayed treatment and elevated risk of transmission [51]. Likewise, vaccine vial shortages in some regions hindered effective and timely vaccination delivery [52]. These circumstances highlight the critical need for more effective procedures and resource allocation to effectively deal with public health emergencies [53]. However, hospital management differs between the public and private sectors. Healthcare today exists as both a system with an imbued right for universal, equitable care governed by legal bodies and as a business, with a private imperative for financial sustainability with a market-driven structure. The recent pandemic demonstrated the need for cross-collaboration, as neither sector is immune to breakdown, but the level of sectoral integration varies worldwide.

In the case of public healthcare systems such as the UK’s National Health Service (NHS) and Canada’s provincial models, the state assumes responsibility for the delivery and financing of core medical services. These models prioritize accessibility but often suffer from rigidity and low surge capacity, hampering the responsiveness of the system, as has been shown in the case of the UK’s NHS [54]. By contrast, private healthcare systems, prevalent in the USA, operate under fee-for-service models that prioritize efficiency, patient choice, and innovation. In private models, fragmentation and financial volatility are a concern. In early 2020, the Centers for Medicare & Medicaid Services (CMS) recommended that most elective surgeries and non-essential medical procedures be cancelled or delayed. Within four months, the American Hospital Association estimated $202.6 billion in losses, or an average of $50.7 billion per month [31]. The sharp decline in revenue for the US hospitals jeopardized providers’ ability to deliver timely and effective care.

**Figure 2:**
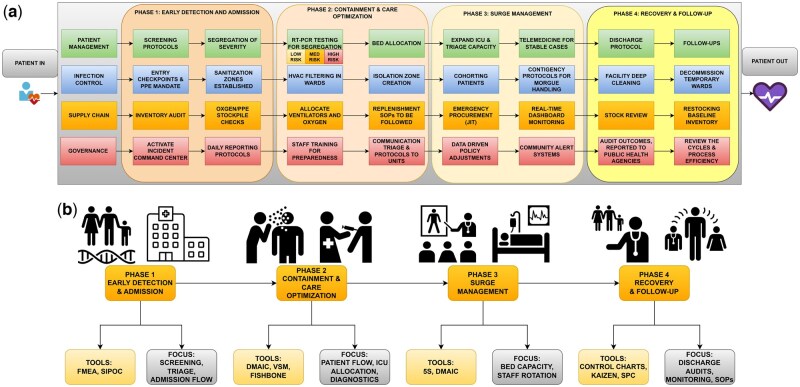
(**a**) Conventional hospital roadmaps for parallel pandemic-centric operations. (**b**) LSS tools and points of focus in each phase of conventional operation.

In the business model dimension, healthcare has increasingly adopted managerial tools from the corporate sector, for example, lean operations, key performance indicators (KPIs), and return-on-investment frameworks. Yet, these tools often emphasize throughput and revenue generation over adaptability and ethics of care. Over-reliance on KPIs can backfire when care quality is hard to quantify. For example, some physicians were penalized for long consultations, even when clinically justified. Performance targets tied to patient turnover led to rushed visits, decreased diagnostic accuracy, and reduced patient satisfaction [54]. Figure 2a comprehensively explains the hospital operations that play a role in pandemic-preparedness. Figure 2b expands on LSS tools that can be used in each phase. Supplementary Appendix B outlines the challenges and possible solutions.

### Policy and implementation strategies: integrating LSS in national healthcare frameworks

Utilizing LSS principles, governments and healthcare sectors can create crisis-ready hospitals. Policy-makers play a vital role in enabling the implementation of LSS; legislation can ensure the smooth introduction of LSS principles into conventional hospital operations. One approach is the provision of regulatory incentives such as tax relief, grants, and direct financial support for organizations that implement LSS methodologies [55]. These financial incentives reward quantitative performance improvements, inspiring continued investment. To prevent incentivization from causing profit-driven attitudes, however, governments should promote holistic policies that discourage over-competition.

Policy-makers can also promote standardization through the enforcement of industry-wide certification programs to enhance credibility and consistency. Universities and medical institutions can offer these certifications in their curricula, ensuring a steady supply of qualified professionals who understand LSS in healthcare contexts [56].

Furthermore, public–private partnerships are critical in advancing LSS usage. To encourage private adoption, governments should first implement LSS in the public sector. Case studies showing the success of LSS in reducing waste and increasing efficiency can serve as models for the private sector. For example, in Italy, a hospital applied LSS to surgical and administrative processes in an effort to decrease the time hip transplant patients spend in the hospital post-procedure. By following DMAIC, patients’ stay was reduced from an average of 18.9–10.6 days [57]. Success stories such as these should inspire the private sector and thereby expand the avenues where LSS can be applied [58].

### Barriers to Lean Six Sigma adoption

There are several barriers that prevent LSS integration. First and foremost, the financial strain during crises forces hospitals to choose between survival and performance enhancements, and funding for projects such as LSS is cut down [59]. Furthermore, both governmental and medical institutions resist major changes. Policy-makers can be held back by path dependency, where past frameworks can hinder new legislation [60]. Healthcare organizations that are accustomed to pre-existing systems are likely to be reluctant to adopt new models. For example, frontline healthcare workers showed reluctance toward implementing new approaches during the pandemic because they believed these alterations would create more problems than solutions and were unable to dedicate time to process improvement [61].

Data inaccuracy is also a significant obstacle. Accurate data is necessary for LSS programs to spot inefficiencies and evaluate outcomes. However, collecting and exchanging vital information becomes increasingly difficult during crises [62]. The use of unreliable data during the pandemic became a barrier as clinicians used systems with non-uniform languages to share information. At the beginning of the pandemic, several Electronic Health Record systems operated without built-in systems to detect COVID-19 patients systematically [63].

Lastly, the complexity of healthcare systems inhibits standardized responses. During the pandemic, hospitals transformed entire administrative units into intensive care units, requiring relocation of staff members [64, 65]. Furthermore, most healthcare leaders did not receive training on crisis management and process improvement techniques [66]. Healthcare leaders centered their pandemic activities on urgent resolution of problems instead of constructing long-term approaches such as LSS [67]. Disruption of medical education and training programs decreased the possibilities for healthcare staff to learn essential LSS skills. Training interruptions affected 81% of healthcare staff, who then became unable to participate in new initiatives [68].

## Discussion

The global health landscape has rapidly evolved over recent decades with the pandemic, climate-related health issues, and antimicrobial resistance threatening systems worldwide. In this context, LSS has the potential to be a transformative tool for enhancing global health security. By facilitating a more structured approach in hospitals and incorporating data-driven strategies, LSS can streamline all aspects of crisis response.

As LSS moves to become a core framework for global health preparedness, it will emphasize real-time surveillance, risk mitigation, and system resilience (Supplementary Appendix C). Tools like value stream mapping and root cause analysis at the population health level can help governments and organizations rapidly detect bottlenecks during crisis response. By integrating LSS with digital health tools like electronic health records and AI-powered decision platforms, the global health community can ensure more agile responses to emerging health concerns.

To address implementation barriers, governments should provide transition assistance, such as training and incentives, to help medical institutions to embrace LSS principles. Furthermore, governments and healthcare systems should continue to emphasize the need for increased pandemic preparedness with empathetic and socioeconomically aware messaging. The future also includes embedding LSS in global health policy planning, such as the WHO’s frameworks, to institutionalize continuous improvement in health governance. To fully harness LSS, health systems must invest in training, capacity-building, and epidemiological risk management, especially in low and middle-income countries where health efficiency can be a key determinant of success.

## Supplementary Material

mzag071_Supplementary_Data

## Data Availability

All relevant data are presented in the manuscript.
