# Supplementary material for: Augmenting healthcare systems for pandemic preparedness: a Lean Six Sigma perspective
Source: Int J Qual Health Care. 2026 May 22;38(2):mzag071. doi: 10.1093/intqhc/mzag071 (PMC13253150; doi:10.1093/intqhc/mzag071)
Supplement: mzag071_Supplementary_Data [file mzag071_supplementary_data.docx]

**Appendix A: Policies During Pandemic**

An examination of existing legislative frameworks, particularly Canada’s Bill C-293 and the WHO’s Pandemic Treaty draft, identifies their response (or lack thereof) to these ethical challenges. The pandemic prompted unprecedented cooperation between governments and pharmaceutical companies, yielding rapid vaccine development. However, it uncovered inequities in resource access and market-driven prioritization. Wealthier nations acquired vaccine stockpiles far in excess of their population needs, while lower-income countries were left waiting, despite WHO’s COVAX initiative [1].

COVID-19 underscored the critical need for robust, adaptable healthcare systems. Adherence to guidelines from WHO, alongside national legislative efforts such as Canada's Bill C-293 and the EU's Digital Health Strategy, is essential for developing transparent, equitable, and ethically sound frameworks for future pandemics [2-4]. Scholarly analyses of pandemic governance further emphasize the need for continuous policy review, adaptive capacity, and ethical oversight in response to evolving public-health challenges. These frameworks must be reviewed and updated regularly to keep up with changing trends in public healthcare policy and their corresponding challenges. **Table A1** comprehensively evaluates the policies implemented during pandemic, with their ethical implications, grey areas, and updates.

**Table A1.** Policies Enforced During the Pandemic and their Implications.

| **Policies During Pandemic** | **Implications for Patient Care** | **Ethical Implications** | **Grey Areas in the Current Policy** | **Addressed in Pandemic Preparedness Documents** | **Ref** |
| --- | --- | --- | --- | --- | --- |
| Resource Allocation Policies (e.g., Ventilator Triage Protocols) | Surge in cases led to shortages of ventilators and ICU beds, forcing triage decisions. | Decisions based on survival probability raised fairness concerns. | Lack of a standardized triage system led to inconsistencies. In the U.S., different states applied different ICU allocation guidelines, causing disparities | BILL C-293 (Canada) mandates the establishment of a federal framework to ensure equitable resource allocation | [5] |
| Visitor Restriction Policies (e.g., No-Visitor) | Strict visitor restrictions protected patients but led to distress. In Los Angeles, patients suffered and died alone due to restrictive hospital policies. | Restricted family presence challenged autonomy and compassion. | Unclear guidelines on compassionate exceptions caused confusion. | The WHO Pandemic Treaty does not define any policy addressing this aspect. | [6, 7] |
| Telehealth Implementation (e.g., Expansion of Virtual Care) | Telehealth expansion ensured continuity of care, but regional disparities in internet accessibility limited use. | Digital divide raised access inequities (rural communities) | Regulatory uncertainty in telehealth reimbursement strategies. | The EU’s Digital Health Strategy includes expanding telehealth infrastructure and subsidies to address digital inequities. | [8, 9] |
| Public Health Mandates (e.g., Mask Mandates, Lockdowns) | Mask mandates and lockdowns helped reduce virus transmission but faced social resistance. | Balancing individual freedoms with public health needs led to debate. | Inconsistent enforcement of mandates.  Experience of different socioeconomic classes. | WHO Pandemic Agreement proposes to standardize international public health mandates. | [10, 11] |
| Vaccination Policies (e.g., Priority Grouping for Vaccines) | Vaccine prioritization aimed to protect high-risk groups, introducing controversial value judgements. | Prioritization created moral dilemmas. | Unclear vaccine prioritization criteria led to public distrust. | BILL C-293 and WHO protocols recommend transparent vaccine prioritization. | [12, 13] |

**Appendix B: Pandemic Operations**

**B.1. Hospital Operations During the Pandemic**

During the pandemic, hospitals encountered unprecedented difficulties in preserving the quality of care, safeguarding healthcare personnel, and reducing mortality rates among patients. To amend these issues, hospitals require effective management. Inefficiencies must be resolved in key areas to enhance patient outcomes and overall hospital efficiency. **Table C1** below outlines the challenges and possible solutions.

**Table B1.** Essential Tasks in the Hospital for Pandemic Operations.

| **Essential Tasks** | **Expected Outcomes** | **Possible Concerns** | **Human Implications due to Inefficiency** | **Effects of Hospital Operations** | **Ref.** |
| --- | --- | --- | --- | --- | --- |
| Covid RT-PCR tests | - Virus not detected - Virus detected - Inconclusive | - False negative reports - False positive reports - Contamination of samples during handling | - Mental trauma - Costs associated with retests - Worsening of health due to delayed or undecisive results | - Improper handling of tests can damage hospital reputation - Possibility of spread of infection to hospital staff and associated losses | [14, 15] |
| Administering vaccines | - Successful administration - Unsuccessful administration | - Improper administration - Insufficient vaccine supply to meet demand - Dosage errors - Contaminated healthcare products | - Side-effects and adverse reactions to vaccines - Mental stress on hospital staff due to workload | - Strain on hospital supply chain - Training costs associated with handling vaccine - Legal liabilities arising from errors associated with vaccine administration | [16, 17] |
| Handling of oxygen cylinders and ventilators | - Synchronised and need based usage of oxygen and ventilators | - Scarcity of oxygen cylinders - Malfunctioning of ventilators and valves - Viral contamination of respiratory aids | - Death of patients due to lack of oxygen supply - Spread of infection through respiratory aids | - Supply chain disruptions associated with oxygen shortage - Potential for reduced capacity due to icing of bulk liquid oxygen systems | [18-20] |
| Patient bed crisis | - Optimized bed utilization - Reduced patient wait times | - Inadequate surge capacity to handle increased patient load - Poor bed management - Lack of coordination between departments | - Increased patient mortality - Increased staff burnout - Delayed or denied care | - Overcrowding in ERs - Cancelled elective surgeries - Damage to hospital reputation - Financial strain | [21-24] |
| Handling of corpses | - Dignified and respectful management of deceased bodies - Prevention of disease spread | - Overwhelmed morgue capacity - Delays in body retrieval - Culturally insensitive body collection processes | - Psychological trauma for survivors - Distress for rescue forces - Violation of cultural norms | - Increased risk of infection spread - Negative impact on community relations - Ethical and legal issues | [25, 26] |

**B.2. Current Influence of LSS in Pandemic Healthcare Operations**

LSS has multidimensional benefits in the healthcare systems ranging from clinical outcomes, operational efficiencies and even organizational impact [27]. There is a plethora of possibilities of LSS application in every aspect of the hospital operations. **Table B2** showcases the benefits of LSS and similar, previously used tools.

**Table B2.** General Influence of LSS in Healthcare.

| **Dimension of Healthcare** | **Advantages** | **Disadvantages** | **Key LSS Tools Used in Research Works** | **Ref** |
| --- | --- | --- | --- | --- |
| Clinical Outcomes | ↓ Errors,  ↓ Infections,  ↓ Readmissions | May overlook patient-centric nuances | DMAIC, SIPOC, Control Charts | [28] |
| Operational Efficiency | ↓ Waste,  ↓ Wait Times,  ↑ Flow | Can be resource-intensive to implement | VSM, 5S | [29, 30] |
| Crisis Response | ↑ Surge Handling,  ↑ Rapid Reconfigurability | Lacks built-in flexibility in legacy models | FMEA, Visual Management | [31, 32] |
| Policy Integration | ↑ Data-Driven Decision-Making | Data-heavy, slow to translate into action | Control Charts, KPIs | [33] |
| System-Wide Scale | ↑ Standardization,  ↑ Cross-team Collaboration | Risk of rigidity and over-standardization | Kaizen, Gemba Walks | [34] |

**Appendix C: Supporting Responsible Innovation with LSS**

In order to promote responsible innovation for the healthcare system, policymakers should facilitate further development of medical technology. The establishment of rapid yet rigorous regulatory approvals for cutting-edge medical technology would significantly expedite their release to market. Expedited pathways would improve a patient’s access to treatment and access to essential resources through resilient supply chains, while maintaining safety standards [35, 36]. Policymakers can also promote interdisciplinary collaboration and increased data transparency between academic and medical institutions [37]. Doing so would encourage up-to-date, widespread knowledge sharing, leading to more informed innovation and enabling enhanced connection between theoretical research and real-world applications.

Outlining clear guidance regarding data privacy and patient safety can build public trust and promote responsible practices. Policymakers should work closely with those within the medical field to develop data-driven policies that balance innovation with consumer protection [35]. By also considering real-world data, policies would be more responsive and effective. Examples include addressing emerging concerns regarding cyber-attacks, such as the use of ransomware to block inter-departmental data sharing. Lastly, establishing controlled testing environments will enable firms to test new technology for product safety and usability. For example, implementing LSS in one department and gradually expanding would build trust in the philosophy and inspire further integration.

**Appendix References**

1. Herzog, L. M., Norheim, O. F., Emanuel, E. J., et al., (2021). Covax must go beyond proportional allocation of covid vaccines to ensure fair and equitable access. Bmj, 372.
2. Parliament of Canada. (2024, June 5). Bill C-293: Pandemic Prevention and Preparedness Act (Third Reading). House of Commons of Canada. <https://www.parl.ca/documentviewer/en/44-1/bill/C-293/third-reading>
3. Evaborhene, N.A., Udokanma, E.E., Adebisi, Y.A., et al., (2023). The pandemic treaty, the pandemic fund, and the global commons: our scepticism. *BMJ Global Health*, *8*(2).
4. Lazarus, J.V., Pujol-Martinez, C., Kopka, C.J., et al., (2024). Implications from COVID-19 for future pandemic global health governance. *Clinical Microbiology and Infection*, *30*(5), pp.576-581.
5. Piscitello, G.M., Kapania, E.M., Miller, W.D., et al., (2020). Variation in ventilator allocation guidelines by US state during the coronavirus disease 2019 pandemic: a systematic review. *JAMA network open*, *3*(6), p.e2012606.
6. Hart, J.L., Turnbull, A.E., Oppenheim, I.M. et al., (2020). Family-centered care during the COVID-19 era. *Journal of pain and symptom management*, *60*(2), pp.e93-e97.
7. Azoulay, E., Cariou, A., Bruneel, F., et al., (2020). Symptoms of anxiety, depression, and peritraumatic dissociation in critical care clinicians managing patients with COVID-19. A cross-sectional study. *American journal of respiratory and critical care medicine*, *202*(10), pp.1388-1398.
8. Mehrotra, A., Chernew, M., Linetsky, D., et al., (2020). The impact of the COVID-19 pandemic on outpatient visits: practices are adapting to the new normal. *The Commonwealth Fund*, *25*, p.2020.
9. Nouri, S., Khoong, E.C., Lyles, C.R., et al., (2020). Addressing equity in telemedicine for chronic disease management during the Covid-19 pandemic. *NEJM Catalyst Innovations in Care Delivery*, *1*(3).
10. Gostin, L.O. and Wiley, L.F., (2020). Governmental public health powers during the COVID-19 pandemic: stay-at-home orders, business closures, and travel restrictions. *Jama*, *323*(21), pp.2137-2138.
11. Bambra, C., Riordan, R., Ford, J. Et al., (2020). The COVID-19 pandemic and health inequalities. *J Epidemiol Community Health*, *74*(11), pp.964-968.
12. National Academies of Sciences, Engineering, and Medicine, 2020. Framework for equitable allocation of COVID-19 vaccine.
13. Persad, G., Peek, M.E. and Emanuel, E.J., 2020. Fairly prioritizing groups for access to COVID-19 vaccines. *Jama*, *324*(16), pp.1601-1602.
14. Public Health Ontario. (2025, April 1). Coronavirus Disease 2019 (COVID-19) – PCR. <https://www.publichealthontario.ca/en/Laboratory-Services/Test-Information-Index/Covid-19>
15. Li, Y., Yao, L., Li, J., et al., (2020). Stability issues of RT‐PCR testing of SARS‐CoV‐2 for hospitalized patients clinically diagnosed with COVID‐19. Journal of medical virology, 92(7), 903-908.
16. Centers for Disease Control and Prevention. (2019, May 7). Preparing vaccines for administration. U.S. Department of Health & Human Services. <https://www.cdc.gov/vaccines/hcp/admin/prepare-vaccines.html>
17. Public Health Agency of Canada. (2023, May). Vaccine administration practices: Canadian Immunization Guide. Government of Canada. <https://www.canada.ca/en/public-health/services/publications/healthy-living/canadian-immunization-guide-part-1-key-immunization-information/page-8-vaccine-administration-practices.html>
18. Lydon, E. (2020, May 29). Bulk medical oxygen systems: How the COVID-19 crisis can impact med-gas infrastructure. Health Facilities Management. <https://www.hfmmagazine.com/articles/3910-bulk-medical-oxygen-systems>
19. Al-Dahhan, W. H., Kadhom, M., Abdallh, M. S., et al., (2022). Medical Oxygen Safe Handling During Coronavirus Pandemic: Short Review. Al-Nahrain Journal of Science, 25(2), 45-50.
20. Bhatt, N., Nepal, S., Pinder, R. J., et al., (2022). Challenges of hospital oxygen management during the COVID-19 pandemic in rural Nepal. The American journal of tropical medicine and hygiene, 106(4), 997.
21. Barchielli, C., Vainieri, M., Seghieri, C., et al., (2023). The Function of Bed Management in Pandemic Times—A Case Study of Reaction Time and Bed Reconversion. International Journal of Environmental Research and Public Health, 20(12), 6179.
22. Pelley, L. (2024). Over-capacity ERs are dangerous choke points. But hospital challenges go far deeper. CBC News. <https://www.cbc.ca/news/health/second-opinion-overcapacity-er-crisis-1.7080946>
23. Fatani, M., Shamayleh, A., & Alshraideh, H. (2024). Assessing the Disruption Impact on Healthcare Delivery. Journal of Primary Care & Community Health, 15, 21501319241260351.
24. Herzlinger, R. E., & Boxer, R. (2022, February 24). Want to prevent the next hospital bed crisis? Enlist the SEC. Harvard Business School Working Knowledge. <https://www.library.hbs.edu/working-knowledge/want-to-prevent-the-next-hospital-bed-crisis-enlist-the-sec>
25. Nejati-Zarnaqi, B., Sahebi, A., & Jahangiri, K. (2021). Factors affecting management of corpses of the confirmed COVID-19 patients during pandemic: A systematic review. Journal of forensic and legal medicine, 84, 102273.
26. Pan American Health Organization. (2020, April 7). Dead body management in the context of the novel coronavirus disease (COVID-19): Interim recommendations. <https://iris.paho.org/bitstream/handle/10665.2/52001/PAHOPHEIHMCovid1920002_eng.pdf>
27. Brandao de Souza, L. (2009). Trends and approaches in lean healthcare. Leadership in health services, 22(2), 121-139.
28. McDermott, O., Antony, J., Bhat, S., et al., (2022). Lean six sigma in healthcare: a systematic literature review on motivations and benefits. *Processes*, *10*(10), p.1910.
29. Kumar, K.S., Babu, R.V. and Paranitharan, K.P., 2022. Application of integrated Lean Six Sigma quality healthcare system practice in Indian healthcare. *International Journal of Value Chain Management*, *13*(1), pp.112-139.
30. Shareifi, D.S.M., Mahawesh, H.E., Alhouri, F.A.A., et al., (2025). Improving Operational Efficiency In Image-Guided Biopsy Clinics: A Lean Six Sigma Approach Integrating Radiology, Nursing, Pharmacy, And Health Services Administration. *The Review of Diabetic Studies*, pp.120-130.
31. Hundal, G.S., Thiyagarajan, S., Alduraibi, M., et al., (2021). Lean Six Sigma as an organizational resilience mechanism in health care during the era of COVID-19. *International Journal of Lean Six Sigma*, *12*(4), pp.762-783.
32. DeRosier, J., Stalhandske, E., Bagian, J.P., et al., (2002). Using health care failure mode and effect analysis™: the VA National Center for Patient Safety’s prospective risk analysis system. *The Joint Commission journal on quality improvement*, *28*(5), pp.248-267.
33. Laney, D. B. (2002). Improved control charts for attributes. Quality Engineering, 14(4), 531-537.
34. Kimsey, D.B., 2010. Lean methodology in health care. *AORN journal*, *92*(1), pp.53-60.
35. Darrow, J. J., Avorn, J., & Kesselheim, A. S. (2021). FDA regulation and approval of medical devices: 1976-2020. Jama, 326(5), 420-432.
36. Ciasullo, M. V., Douglas, A., Romeo, E., et al. (2024). Lean Six Sigma and quality performance in Italian public and private hospitals: a gender perspective. International Journal of Quality & Reliability Management, 41(3), 964-989.
37. Ivankovic, D., Garel, P., Klazinga, N. Et al., (2023). Data-driven collaboration between hospitals and other healthcare organisations in Europe during the COVID-19 pandemic: An explanatory sequential mixed-methods study among mid-level hospital managers. *International journal of integrated care*, *23*(2), p.28.
